# Circulação Extracorpórea na Cirurgia de Revascularização do Miocárdio no Estado de São Paulo. O Estudo REPLICCAR

**DOI:** 10.36660/abc.20200914

**Published:** 2020-10-13

**Affiliations:** 1 Universidade de São Paulo Faculdade de Medicina de Ribeirão Preto Departamento de Cirurgia e Anatomia São PauloSP Brasil Departamento de Cirurgia e Anatomia, Faculdade de Medicina de Ribeirão Preto, Universidade de São Paulo, São Paulo, SP - Brasil

**Keywords:** Infarto do Miocárdio/cirurgia, Revascularização Miocárdica/cirurgia, Circulação Extracorpórea, Epidemiologia, REPICCAR

Se a revascularização do miocárdio deve ser realizada com ou sem o uso de circulação extracorpórea, chamada de CRM sem CEC e com CEC, ainda é uma questão em debate. Intuitivamente, evitar o *bypass* cardiopulmonar parece benéfico, pois a resposta inflamatória sistêmica da circulação extracorpórea é omitida. Mesmo assim, nenhum ensaio randomizado único foi capaz de provar que a CRM sem CEC é superior à CRM com CEC no que diz respeito aos desfechos de morte, acidente vascular cerebral ou infarto do miocárdio.

Hoje em dia, a cirurgia de revascularização do miocárdio sem CEC (RMSCEC) se tornou uma prática comum na cirurgia de revascularização do miocárdio (CRM). Além disso, parece que disfunções de órgãos (fígado, rim, isquemia intestinal, acidente vascular cerebral e outros tipos de disfunções menores) devem ser definitivamente diferenciadas, considerando as duas cirurgias de revascularização do miocárdio. Uma limitação associada à técnica sem CEC, especificamente a instabilidade hemodinâmica, diz respeito à qualidade da anastomose, a capacidade de obter uma revascularização completa e a taxa de conversão para CEC, que são preocupações especulativas. Portanto, ainda não está claro se a RMSCEC é superior em termos de patência do enxerto, incidência de complicações, desfechos em longo prazo e a taxa de mortalidade associada em comparação com a CRM convencional (CRMC).

O boletim da Sociedade Brasileira de Cirurgia Cardiovascular (SBCCV) (abril de 2017) destaca um consenso publicado pela *American Heart Association* (AHA) para o uso de critérios adequados para revascularização miocárdica em angina estável.^1^ Diferentemente de uma diretriz padrão, este Consenso traz mais de 60 cenários clínicos reais, avaliados por um painel de 32 especialistas entre médicos, intervencionistas e cirurgiões. Foram contempladas as características clínicas, anatômicas e funcionais e, de forma inovadora, o tratamento com um ou mais antianginosos pesou na decisão da intervenção. Essa abordagem tem sido útil para estabelecer uma padronização inequívoca para corrigir discrepâncias regionais quando, por exemplo, o EuroSCORE e STS são utilizados. A cirurgia cardíaca brasileira, embora tenha alto prestígio internacional, nunca realizou um grande “ensaio” sobre a cirurgia de revascularização do miocárdio sem circulação extracorpórea, já que sua introdução na prática cirúrgica foi realizada pelo Dr. Enio Buffolo (no Brasil) e Dr. Federico Benetti (na Argentina).^2^

Pelo menos dois ensaios em andamento (BYPASS REGISTRY e REPLICCAR) poderiam se tornar um valioso ponto de partida para o real estabelecimento das condições da cirurgia cardíaca no Brasil. O projeto BYPASS está ganhando corpo e cumprindo o objetivo de retratar o cenário da cirurgia cardiovascular no Brasil.^3^^,^^4^ Apesar de várias tentativas anteriores de estabelecer um banco de dados nacional, este projeto merece ser incentivado. Esses dois estudos devem ser o início de um banco de dados unificado da cirurgia cardíaca brasileira.

Nesta edição dos Arquivos Brasileiros de Cardiologia, temos o prazer de ler os primeiros resultados do estudo REPLICCAR, que considerou dados extraídos de instituições acadêmicas do estado de São Paulo. Os desfechos analisados foram: morbidade (reoperação por sangramento, choque cardiogênico, acidente vascular cerebral, infecção do sítio cirúrgico, mediastinite, pneumonia, infarto do miocárdio, insuficiência renal aguda e mortalidade cirúrgica no período entre a cirurgia e a avaliação de 30 dias, ou até a alta hospitalar. O estudo enfatiza que, embora existam critérios bem definidos para a indicação de CRM, a escolha da CEC permanece baseada no perfil clínico do paciente e na experiência do cirurgião. No estudo REPLICCAR, a reoperação por hemorragia foi o único desfecho associado ao uso de CEC na CRM.^5^

Por fim, qual é a melhor resposta à pergunta desafiadora a qual é sempre repetida sobre as duas técnicas? a RMSCEC é melhor do que a CRM convencional ou vice-versa? Sim ou não? Os estudos REPLICCAR e BYPASS não ajudaram e, por enquanto, a resposta mais segura para a pergunta continua sendo “TALVEZ” … Intuitivamente, temos a impressão de que, após mais de 30 anos, ambas as técnicas encontraram seu lugar, incluindo o aumento de “enxertos híbridos”, os quais seriam estudados em parceria com os dois estudos acima mencionados.

## References

[1] 1. Patel MR, Calhoon JH, Dehmer GJ, Grantham JA, Maddox TM, Maron DJ, et al. J Am Coll Cardiol. ACC/AATS/AHA/ASE/ASNC/SCAI/SCCT/STS 2017 Appropriate use criteria for coronary revascularization in patients with stable ischemic heart disease: a . J Am Coll Cardiol. 2017;69(17):2212–41. 10.1016/j.jacc.2017.02.001 28291663

[2] 2. Braile DM, Évora PRB. The Brazilian Cardiac Surgery, Although it has High International Prestige, Never Performed a Great “Trial”. Braz J Cardiovasc Surg. 2017;32(6):I-II. 10.21470/1678-9741-2017-0509 PMC5731304 29267605

[3] 3. Paez RP, Hossne Junior NA, Espirito Santo JA, Santos R, Kalil R. Jatene F, et al. Coronary Artery Bypass Surgery in Brazil: Analysis of the National Reality Through the BYPASS Registry. Braz J Cardiovasc Surg. 2019;34(2):142-8. 10.21470/1678-9741-2018-0313 PMC6436784 30916123

[4] 4. Dallan LAO. Comment on the study Coronary Artery Bypass Surgery in Brazil: Analysis of the National Reality Through the Bypass Registry that was presented at the 46^th^ Congress of the Brazilian Society of Cardiovascular Surgery, Nova Lima, BH, Brazil, April 5 and 6, 2019. Braz J Cardiovasc Surg. 2019;34(4):504-6. 10.21470/1678-9741-2019-0606 PMC6713380 31454210

[5] 5. Borgomoni GB, Mejia OAV, Orlandi BMM, Lisboa LAF, Conte PH, Oliveira MA et al, Current Impact of Cardiopulmonary Bypass in Coronary Artery Bypass Grafting in São Paulo State. Arq Bras Cardiol. 2020; 115(4):595-601. 10.36660/abc.20190145 PMC8386981 33111853

