# Supplementary material for: Circulação Extracorpórea na Cirurgia de Revascularização do Miocárdio no Estado de São Paulo. O Estudo REPLICCAR
Source: Arq Bras Cardiol. 2020 Oct 13;115(4):602–3. [Article in Portuguese] doi: 10.36660/abc.20200914 (PMC8386963; doi:10.36660/abc.20200914)
Supplement: Translation

# Translation: Cardiopulmonary bypass in Myocardial Revascularization Surgery in the State of São Paulo. The REPLICCAR Study

**Affiliations:** 1 São PauloSP Brazil Departamento de Cirurgia e Anatomia, Faculdade de Medicina de Ribeirão Preto, Universidade de São Paulo, São Paulo, SP - Brazil

**Keywords:** Myocardial Infarction/surgery, Myocardial Revascularization/surgery, Extracorporeal Circulation, Epidemioloy, REPICCAR

Whether myocardial revascularization should be performed with or without the use of cardiopulmonary bypass, referred to as off-pump and on-pump CABG, is still up for debate. Intuitively, avoidance of cardiopulmonary bypass seems beneficial, as the systemic inflammatory response from extracorporeal circulation is omitted. Even so, no single randomized trial has been able to prove that off-pump CABG is superior to on-pump CABG, as regards the hard outcomes of death, stroke or myocardial infarction.

Nowadays, Off-pump coronary artery bypass grafting (OPCAB) has become a common practice for coronary artery bypass grafting (CABG). Also, it seems that organ dysfunctions (liver, kidney, bowel ischemia, stroke and other kinds of minor dysfunctions) should be definitively differentiated, considering the two myocardial revascularization operations. A limitation associated with the off-pump technique, namely hemodynamic instability, concerns the quality of the anastomosis, the ability to achieve complete revascularization and the on-pump conversion rate, constituting speculative concerns. Therefore, it is still unclear whether OPCAB is superior in terms of graft patency, the incidence of complications, long-term outcomes, and the associated mortality rate- compared with conventional CABG (CCABG).

The Brazilian Society of Cardiovascular Surgery (SBCCV) newsletter (April 2017) highlights a consensus published by the American Heart Association (AHA) for the use of appropriate criteria for myocardial revascularization in stable angina.^1^ Unlike a standard guideline, this Consensus brings more than 60 real clinical settings, scored by a panel of 32 experts among clinicians, interventionists, and surgeons. The clinical, anatomical and functional characteristics were contemplated, and innovatively, the treatment with one or more antianginal drugs weighed in the intervention decision. This approach has been useful to establish an unambiguous standardization to correct regional discrepancies when, for example, EuroSCORE and STS are used. The Brazilian cardiac surgery, although it has high international prestige, never performed a great “trial” about coronary artery bypass grafting without cardiopulmonary bypass, since its introduction in surgical practice was carried out by Dr Enio Buffolo (in Brazil) and Dr Federico Benetti (in Argentina).^2^

At least two Trials in progress (BYPASS REGISTRY and the REPLICCAR) could become a valuable starting point for the real establishment of cardiac surgery conditions in Brazil. The BYPASS project is taking shape and accomplishing the aim of providing a picture of the Brazilian cardiovascular surgery scenario.^3^^,^^4^ Despite several previous attempts to establish a national database, this project deserves to be incentivized. These two studies should be the beginning of a unified Brazilian cardiac surgery database.

In this *Arquivos Brasileiros de Cardiologia* issue, we have the pleasure to read the initial results of the REPLICCAR study, which considered data extracted from academic institutions from the state of São Paulo. The analyzed outcomes were: morbidity (reoperation due to bleeding, cardiogenic shock, stroke, surgical site infection, mediastinitis, pneumonia, myocardial infarction, acute renal failure and surgical mortality in the period between the surgery and the 30-day evaluation, or until hospital discharge. The study emphasizes that, although there are well-defined criteria for CABG indication, the choice of CPB remains based on the patient's clinical profile and the surgeon's experience. In the REPLICCAR, bleeding reoperation was the only outcome associated with the use of CPB in CABG.^5^

Finally, what is the best answer to the challenging question that is always repeated about the two techniques? Is the OPCAB better than the conventional CABG (CCABG) or vice versa? Yes or no? REPLICCAR and BYPASS studies were not helpful in answering that, and for now, the safest answer to the question remains “MAYBE”… Intuitively, one has the impression that, after more than 30 years, both techniques have found their place, including the increase in “hybrid grafts”, which would be studied in partnership with the two aforementioned studies.
